# Cement-Based Solidification/Stabilization as a Pathway for Encapsulating Palm Oil Residual Biomass Post Heavy Metal Adsorption

**DOI:** 10.3390/ma15155226

**Published:** 2022-07-28

**Authors:** Candelaria Tejada-Tovar, Angel Villabona-Ortíz, Ángel González-Delgado

**Affiliations:** 1 Process Design and Biomass Utilization Research Group (IDAB), Chemical Engineering Department, Universidad de Cartagena, Avenida del Consulado St. 30, Cartagena de Indias 130015, Colombia; avillabonao@unicartagena.edu.co; 2 Nanomaterials and Computer-Aided Process Engineering Research Group (NIPAC), Chemical Engineering Department, Universidad de Cartagena, Avenida del Consulado St. 30, Cartagena de Indias 130015, Colombia; agonzalezd1@unicartagena.edu.co

**Keywords:** immobilization, cement, adsorption, biomass, heavy metal

## Abstract

Heavy metal pollution is a serious issue currently affecting the environment and public health, which has been faced by applying several alternatives such as adsorption. In this work, the adsorption technique was employed to remove nickel and lead ions from an aqueous solution using palm oil residual biomass as a biosorbent. Desorption experiments were also conducted to evaluate the desorption capacity of this biomass over sorption–desorption cycles. The polluted biomass was used to prepare bricks (5 and 10% biomass content) to encapsulate heavy metal ions into the cement matrix. Both mechanical resistance and leaching testing were performed to determine the suitability of these bricks for construction applications. The experimental results revealed a good biosorbent dosage of 0.1 g/L. The highest desorption yields were calculated in 11 and 83.13% for nickel and lead, respectively. The compression resistance when 10% biomass was incorporated into the bricks was reported to be below the acceptable limit. Leaching testing suggested a successful immobilization of heavy metal ions onto the cement matrix. These results indicate that the application of this immobilization technique allows solving disposal problems of biomass loaded with heavy metal ions.

## 1. Introduction

The rapid growth of the population worldwide has contributed to an increase in several environmental problems as heavy metal pollution, which affects the survival of all living organisms and can have several adverse effects on the human body [1,2]. Heavy metals are characterized as not biodegradable and can accumulate in organisms producing toxic and carcinogenic effects [3]. These pollutants are discharged into the environment due to anthropogenic activities as smelting, combustion of leaded gasoline, and fertilizers to land [4].

Lead is considered one of the most phytotoxic metals causing cell membrane damage, severe infections in digestive organs, and reproductive disorders [5,6]. Aqueous residues of these heavy metal ions are generated mainly by battery and metal plating facilities [7]. Nickel also produces acute effects on organisms such as nausea, chest pain, dizziness, and cyanosis [8]. Several alternatives have been developed for the removal of these pollutants from wastewater including precipitation, ion exchange, coagulation, evaporation, and reverse osmosis [9]. However, these techniques have limited application because of the high sludge volume, high costs, and extended times [10]. To face these disadvantages, the adsorption technique is widely employed because of its high efficiency, ease of operation, little sludge generation, and low cost [11].

Many low-cost materials have been used in nickel and lead removal from wastewaters, among these, agro-industrial biomass seems to be a promising source of biosorbent owing to its availability [12]. The oil palm industry produces a large number of wastes such as fresh fruit bunch, oil palm fiber, and empty fruit bunches, which are sources of biomass [13]. Due to the 60% increase in oil production forward from oil palm, about 500,000 tons of waste are generated per year [14]. For all published cases, the final destination of the new waste (biomass + pollutant) remains a problem, and the question arises as to whether a new waste is being generated that is also harmful to the environment.

After adsorption application, these biomaterials loaded with heavy metal ions require further disposal and stabilization/solidification with cement-based binders (S/S) as an option to immobilize them [15]. The cement matrix stabilizes and immobilizes the pollutants by solidification [16]. Recently, Villabona-Ortiz et al. [17] used this technique to encapsulate lead and nickel ions in cassava peel biomass and reported promising mechanical results for bricks and low concentrations of leachate during tests. During encapsulation, heavy metal ions are sequestered in their crystalline form, forming, for example, phases similar to zeolites and capillary pores, which reduces the activity and solubility of heavy metals [18].

In this work, palm oil residual biomass was used for removing lead and nickel from an aqueous solution. The desorption capacity of this material was also evaluated via sorption–desorption cycles. The cement-based stabilization/solidification technique was applied by preparing bricks with the polluted biomass. In this sense, encapsulation of highly contaminated biomass with heavy metal ions was selected as a suitable alternative to solve disposal problems of the adsorption technique.

## 2. Materials and Methods

### 2.1. Biomass Preparation

Residual biomass from palm oil crops was used as a biosorbent because of its availability and adsorption properties. The palm oil fruit shells were prepared by adding water to remove surface-adhered particles [19]. The biomass was dried, grounded, and sieve-meshed to achieve the particle size selected for performing the adsorption process (1 mm). To characterize the biomass before and after the adsorption process, the Perkin Elmer (Waltham, MA, USA) Fourier-Transform Infrared Spectroscopy (FTIR) model 1600 was used making 128 sweeps in the range of 400–4000 cm^−^¹.

### 2.2. Sorption-Desorption Cycles

The adsorption capacity of the palm oil fruit shells was determined by carrying out batch adsorption experiments using nickel and lead to preparing the polluted solutions. As is well known, several factors affect the adsorption process such as temperature, pH, adsorbent dosage, and stirring speed, hence, they were fixed at 40 °C, 6, 0.5 g/L, and 200 rpm, respectively. The solutions of heavy metals were prepared by adding Pb(NO_3_)_2_ and Ni(NO_3_)_2_ into deionized water, at an initial concentration of 100 mg/L. An atomic absorption spectrophotometer was used to measure the remaining concentration of heavy metal after completing the contact time between biomass and polluted solution [19]. Metals were detected using a Thermo Fisher^®^ (Waltham, MA, USA) atomic absorption spectrophotometer with Solar S4 flame (Waltham, MA, USA), with a detection limit of 0.05 mg/L. For Nickel (II) a Nickel hollow cathode lamp was used at 232 nm; Lead was detected at 217 nm in the atomic absorber equipped with single element hollow cathode lamps and an air–acetylene burner was used for the determination.

The adsorption process causes biomass saturation with metallic ions; hence, it was required to filter and weigh the biosorbent before each desorption cycle. Two different types of acids (hydrochloric and nitric) were selected as desorbing agents and the effect of their concentrations was also evaluated. The biomass regeneration was performed by contacting the palm oil fruit shells with calcium chloride at 4 °C [20].

### 2.3. Cement-Based Solidification/Stabilization

The concrete matrix was composed of: 18.1% cement, 30.6% sands, 43.2% grit, and 8.1% water [21]. Different amounts of polluted biomass were selected to be added to the whole mixture prepared for manufacturing of the concrete matrix (5 and 10% wt). Then, the bricks were formed by hardening the resulting mixture for 28 days with dimensions measuring 5 cm × 5 cm × 5 cm [22].

### 2.4. Mechanical Resistance Testing

Compression resistance testing was performed using a digital universal testing machine TINIUS OLSEN to study the capacity of bricks to resist axial forces until failure, according to the ASTM C109 standard [22]. During the test, an axial charge was applied to the concrete cube from 0 MPa until failure occurred, the electronic board measured the force that was applied until the cube ruptured.

### 2.5. Leaching Tests

The leaching tests involved sample collecting and mixing with water as reported by Ukwatta et al. [23]. After adjusting pH, the mixture was heated for 10 min and cooled to measure the final pH, which was expected to be below 5.

## 3. Results

### 3.1. Biomass Characterization

**Figure 1:**
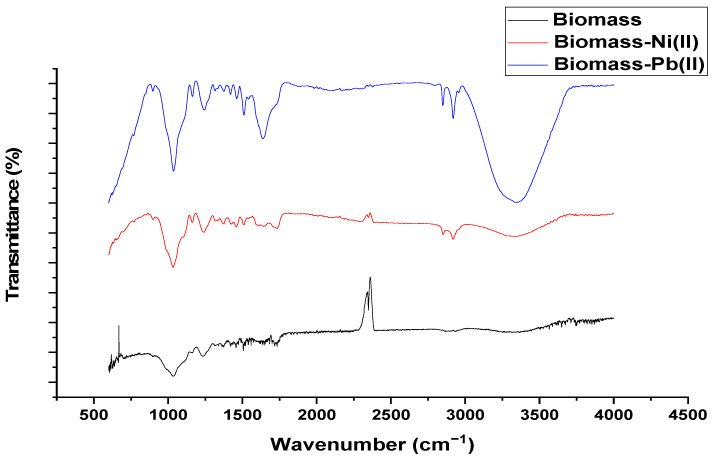
FT-IR spectrum of palm oil fruit shells before and after Pb (II) and Ni (II) uptake.

Figure 1 shows the FT-IR spectrum for palm oil fruit shells before and after the adsorption process.

Results from the conducted FT-IR show the presence of an absorbance band attributed to stretching vibrations of hydroxyl groups at 3367 cm^−1^ [24]. The carbonyl group in lignin composition was identified around 1600 and 1715 cm^−1^ [5]. The peaks at 1450 and 1540 cm^−1^ were assigned to aromatic, aliphatic, and methoxy groups [25]. The stretching vibrations of alcohol and carboxylic acid groups were found around 1050 and 1450 cm^−1^ [26]. The spectrum of biomass polluted with lead and nickel showed that hydroxyl and carboxylic acid groups contribute most to the adsorption process because of the stretching of these functional groups around 3400 and 2360 cm^−1^.

### 3.2. Effect of Biosorbent Dosage

**Figure 2:**
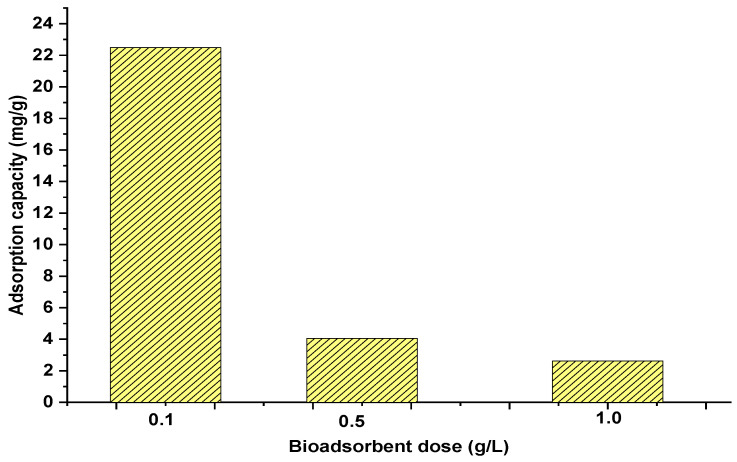
Effect of biosorbent dosage on adsorption capacity for nickel uptake.

Batch adsorption experiments were performed to determine the adsorption capacity of palm oil fruit shells for uptake of heavy metal ions. The effect of biosorbent dosage was evaluated to select the most suitable value for conducting further experiments. Figure 2 shows that biosorbent dosage significantly affected the adsorption process after 24 h of contact time. An increase in adsorption capacity was found as the biosorbent dosage decreased due to the biomass agglomeration. The highest adsorption capacity of 22.49 mg/g was achieved using a dosage of 0.1 g/L, which can be attributed to physical adsorption affecting nickel and lead uptake [27].

**Figure 3:**
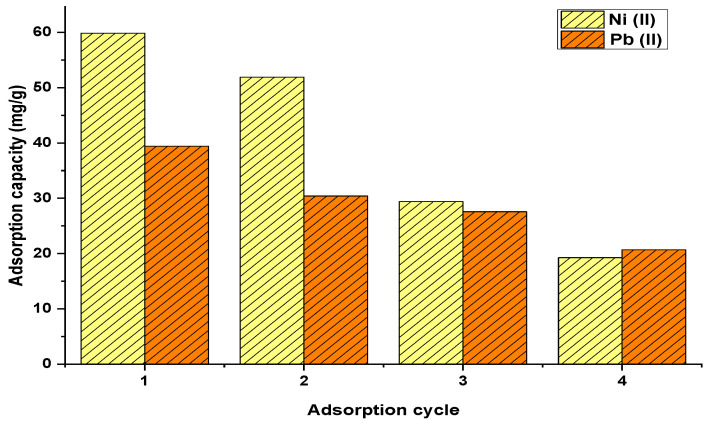
Adsorption cycles for nickel and lead uptake using palm oil fruit shells.

The adsorption cycles using the palm oil fruit shells are shown in Figure 3. Nickel ions were more absorbed by this biomass than lead ions. The adsorption capacities for nickel and lead ions measured up to 58 and 39 mg/g, respectively. The biomass exhibited a reduction in adsorption affinity over the desorption cycles, which can be attributed to damage in the active sites of the biosorbent generated by desorbing agent [28]. Tejada-Tovar et al. [29] pointed out that the adsorption process is enhanced by stretching of absorption bands assigned to hydroxyl and carbonyl groups.

### 3.3. Desorption Experiments

#### 3.3.1. Effect of Desorbing Agent

**Figure 4:**
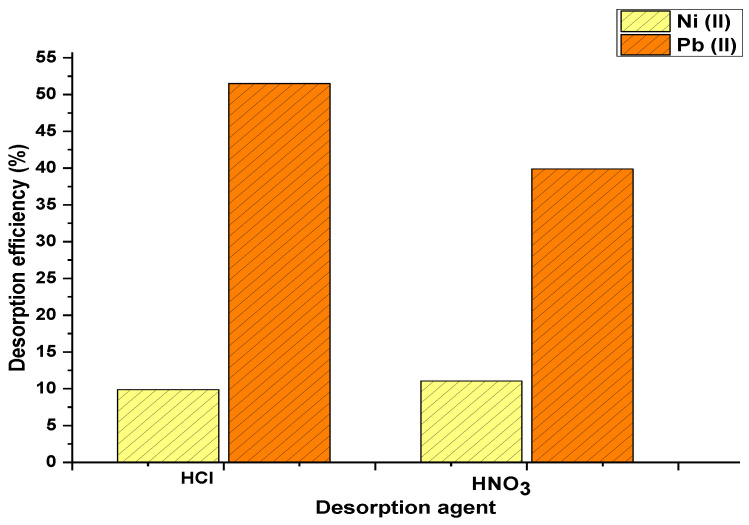
Effect of desorbing agent type on desorption yield.

Figure 4 shows the influence of desorbing agent type on the desorption process using palm oil fruit shells as biosorbent for nickel and lead uptake. It was found that the type of desorbing agent significantly affected desorption yield. Nickel reached highest adsorption yields when the desorption agent was nitric acid; on the other hand, lead achieved the best results when using hydrochloric acid. Desorption of heavy metal ions could occur by the ionic exchange between protons in the desorbing agent and metallic cations previously adsorbed. Lezcano et al. [28] performed desorption experiments using HCl and NaHCO_3_ for removing lead from a biomass of a eutrophic habitat and found higher desorption yields using HCl.

**Figure 5:**
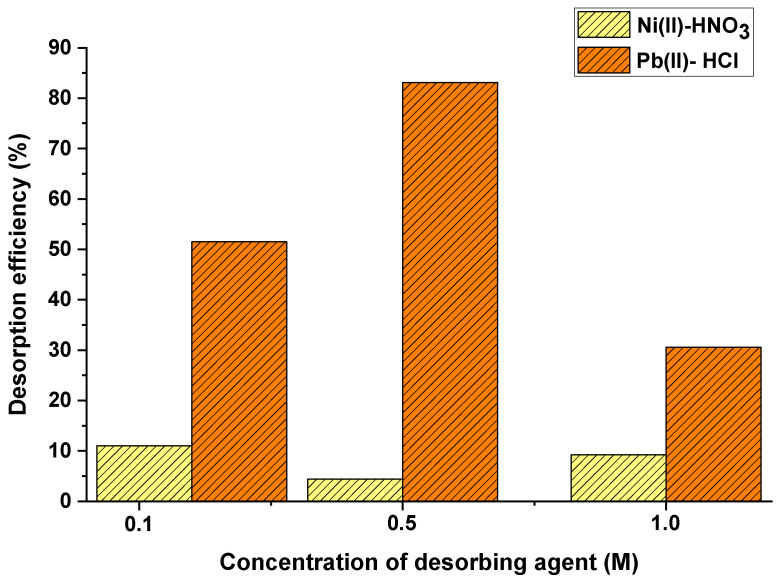
Effect of desorbing agent concentration on nickel and lead desorption yield.

After selecting the desorbing agent type for each heavy metal, the effect of its concentration was also evaluated. As shown in Figure 5, the nitric acid concentration of 0.1 M reached the highest desorption yield for nickel (11%). The results indicate that the desorption efficiency of the oil palm bagasse is higher at the lowest HNO_3_ concentration evaluated and then decreased with increasing HNO_3_ concentration. This observation is likely attributable to the fact that a higher concentration of HNO3 would dramatically increase the H^+^ concentration in solution and thus may enhance electrostatic repulsion among Ni(II) ions, inhibiting Ni(II) desorption [30].

The lead desorption process indicated that hydrochloric acid at 0.5 M solution allowed obtainment of the highest desorption yield (83.13%). These results are similar to those obtained by Elgarahy et al. [31], who reported desorption yields up to 45% attributed to the amount of acid used in desorption experiments. HCl and HNO_3_ were tried in the present study because desorption of heavy metals, under acidic conditions causes protonation of the sorbent surface which allows desorption of positively charged metal ions from adsorbent [32]. The increase in acid concentration leads to the accumulation of H+ in solution, thus increasing the concentration gradient of ions and H+ and resulting in an increased driving force for ion-exchange, favoring the desorption process. However, too high concentrations might cause the destruction of the active adsorption sites [33].

#### 3.3.2. Desorption Cycles

**Figure 6:**
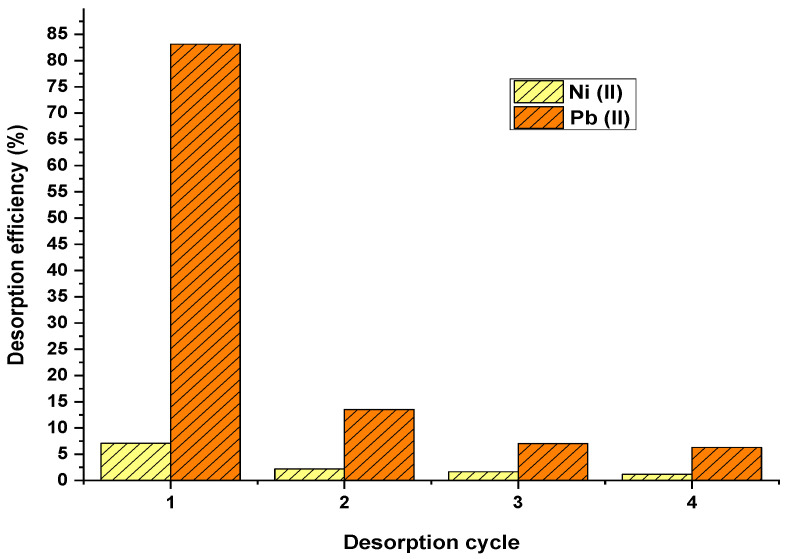
Performance of biomass over desorption cycles.

The palm oil fruit shells saturated with nickel ions exhibited lower desorption capacity than lead as shown in Figure 6. During the first cycle, the desorption yields achieved were 7.10 and 83.13% for nickel and lead ions, respectively. As the number of cycles increased, the desorption yields reduced, which can be attributed to damage in biomass structure after using desorbing agents [34].

### 3.4. Mechanical Resistance Testing

**Figure 7:**
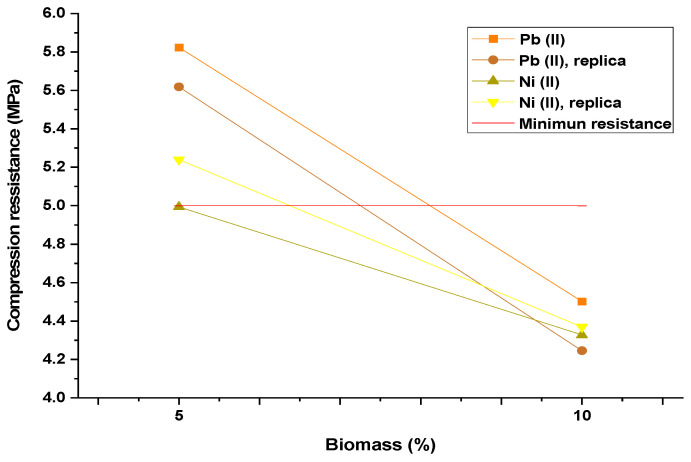
Effect of biomass on the mechanical compression resistance of bricks.

The mechanical resistance testing indicated that the bricks prepared by adding biomass polluted with nickel ions exhibited the lowest resistance. The compressive strength of the mixture without adding biomass with heavy metals was 5.6 MPa. As shown in Figure 7, for both heavy metal ions, the bricks with 10% biomass showed compression resistance below the acceptable limit stablished in according to the NTC 220 [35] equivalent to a load of 12.5 kN for a brick of 25 cm^2^; which can be attributed to the presence of spacing between biomass and cement matrix.

The degradation of biomass after contact with alkaline agents generated during pozzolanic reactions in concrete production and in the cement can also affect the compression resistance [36]. Moreover, the porosity of the organic materials, such as the oil palm residue, causes the mixture to absorb water when it is prepared and the mixture remains drier, thus the pozzolanic reactions that produce the formation of CaH_2_SiO_4_ 2H_2_O (CSH gel), which determines the workability, consistency and mechanical properties of the hardened concrete matrix and is generated forward from the reaction of CaO and SiO_2_ of the concrete in the presence of water, would not develop correctly [37]. Hence, it is recommended not to use this mixture composition for preparing bricks without previous pretreatment of the biomass.

**Figure 8:**
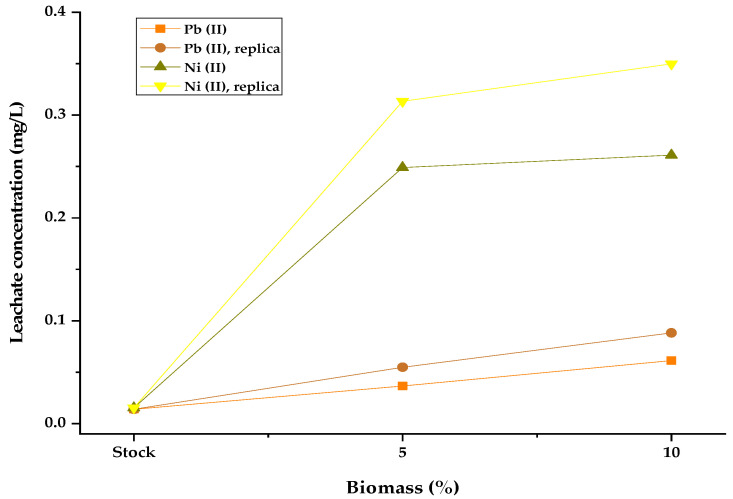
Leaching tests for bricks prepared with polluted biomass.

As seen in Figure 8, it is established that a lower resistance is achieved with nickel at 5.19 MPa, while lead resistance of 5.41 MPa is achieved. This is because Pb (II) has a higher affinity to concrete. Similar results were found when using bagasse ash, detecting Pb(II) concentrations <0.02 and <0.001 after 7 and 28 days of curing, respectively [38]. Regarding the biomass–cement ratio, resistance decreases when the percentage of biomass in the mixture increases, owing to the decrease in cement used and hydration of the mixture, causing less C-S-H generation [39].

### 3.5. Leaching Testing

This testing is important to be performed because of the environmental regulations related to the concentration of heavy metal ions in the leachate. Table 1 summarizes the results of leaching testing. It was found that the biomass polluted with nickel and lead is leached producing leachate with heavy metal concentrations below the regulation (Decree 1594/1984).

As shown in Figure 8, the highest leachate concentrations were 0.08 and 0.36 mg/L for lead and nickel, respectively, which suggested that the heavy metals were successfully encapsulated in the solidified concrete matrix. These results could be attributed to the formation of bonds between heavy metals and oxides found in the cement as CaO, Al_2_O_3,_ and Fe_2_O_3_ [40].

From the results, it is established that increasing the biomass content in the block increases the concentration of heavy metals in the leachate. This is possibly caused by the fact that organic materials can decrease the strength of the concrete, due to lower compaction of the mixture because of the decrease in the formation of the calcium silicate gel (C-S-H), a product of the pozzolanic reactions that occur during the curing of the concrete with Portland cement; thus, the lower the compaction, the higher the leaching of the metal [37]. Since the formation of C-S-H allows the adsorption and absorption of ions; a decrease in alkalinity may also occur, which favors the precipitation of insoluble hydroxides [41]. Similarly, the higher the percentage of biomass in the mixture, the higher the ratio of retained ions, which increases the leaching of ions. On the other hand, Ni(II) is the metal that leaches the most, because it adheres to the active sites of the biomass in a lower proportion than Pb(II), and therefore, detaches more easily from the cement matrix [42]. The results obtained in the leaching tests on the concrete blocks are consistent with the compressive strength tests, taking into account the decrease in mechanical properties due to the presence of biomass and heavy metals in the cement matrix.

## 4. Conclusions

This work attempted to apply cement-based solidification/stabilization as an immobilization technique for encapsulating Ni (II) and Pb (II). Residual biomass from the palm oil industry was selected as biosorbent and characterized by FT-IR analysis. The spectrum confirmed the presence of hydroxyl and carbonyl groups in the palm oil fruit shells, which contribute significantly during the adsorption process. It was found that the highest adsorption capacity was achieved using a biosorbent dosage of 0.1 g/L. Better results for adsorption capacities were reported for nickel than lead. Regarding desorption experiments, the highest desorption yield was reached using 0.1 M nitric acid and 0.5 M hydrochloric acid for nickel and lead, respectively. The mechanical resistance and leaching test, suggest that increasing the amount of biomass decreases the compressive strength and the concentration of heavy metals in the leachate, 10% biomass bricks could not resist compression until the acceptable limit. The leachate concentration results suggested the successful encapsulation of heavy metal ions obeying environmental regulation. Finally, it can be concluded that cement-based solidification/stabilization provides a promising alternative to face the environmental issue of heavy metal pollution.

## Figures and Tables

**Table 1:** Leaching test results.

Brick Composition	Leachate (mg/L)	Environmental Regulation
5% Biomass-Pb (II)	0.0365653	0.5
10% Biomass-Pb (II)	0.0613582	0.5
5% Biomass-Pb (II), replica	0.0548105	0.5
10% Biomass-Pb (II), replica	0.0882052	0.5
5% Biomass-Ni (II)	0.2489509	2
10% Biomass-Ni (II)	0.2611262	2
5% Biomass-Ni (II), replica	0.3134346	2
10% Biomass-Ni (II), replica	0.3498111	2

## Data Availability

The data that support the findings of this study are available from the corresponding author, upon reasonable request.
